# Validation of a Virtual Reality-Based Timed Up-and-Go Test Using Body-Worn Motion Trackers

**DOI:** 10.3390/s26123669

**Published:** 2026-06-08

**Authors:** Brooke E. Peters, Sherry Law, Simon Dugré-Rezun, Alana Gullison, Chris A. McGibbon

**Affiliations:** 1 Faculty of Kinesiology, University of New Brunswick, Fredericton, NB E3B 5A3, Canada; 2 Innerva Virtual, Inc., Fredericton, NB E3C 0J1, Canada; sherry.law87@gmail.com (S.L.);; 3 Institute of Biomedical Engineering, University of New Brunswick, Fredericton, NB E3B 5A3, Canada

**Keywords:** head-mounted virtual reality, body-worn motion tracking, clinical tests of mobility and balance, affordable and accessible motion capture

## Abstract

**Highlights:**

**What are the main findings?**

- Virtual timed-up-and-go (TUG) was functionally equivalent to the standard TUG in a cohort of young and older healthy adults.
- Automated TUG sub-task times (chair, walk, turn) were quantified from body-worn motion trackers.
- The virtual TUG was safe, and user satisfaction was high.

**What are the implications of the main findings?**

- Body-worn motion trackers with head-mounted VR add considerable value to common assessments of physical functioning such as the TUG test.
- Normative values for the virtual TUG test may still be required.
- Standardized protocols for VR-based motion capture are warranted.

**Abstract:**

Background: Full-body motion capture using commercial virtual reality (VR) systems offers unique opportunities for augmenting common functional assessment such as the timed up-and-go (TUG) test. The purpose of this study was to determine whether task performance (chair, walk, turns) during a VR version (vTUG) is parametrically equivalent to the standard test (sTUG). Methods: Twenty healthy adult participants (age 19–71 years) were evaluated with the sTUG followed by the vTUG version of the same test. Body trackers were used to capture kinematics during both tests. TUG time was measured manually with a stopwatch. Tracker data were used to automatically quantify total TUG time and sub-task times for chair, walk and turn portions. Absolute agreement was evaluated using Intraclass Correlation Coefficient (ICC(2,k)) and Bland–Altman analysis. A custom survey was used to evaluate user satisfaction. Results: Very good agreement (ICC > 0.8) was found between sTUG and vTUG for manual and automated measures of total time. ICCs for sub-task times were acceptable (ICC > 0.7) for chair rise, walks and first turn but less so for second turn and sit (ICC < 0.7). User satisfaction was high, and there were no adverse events. Interpretation: The vTUG and sTUG are parametrically equivalent, though sub-task segmentation may require more research. Nevertheless, VR body trackers are a value-added feature whether used with the vTUG or the sTUG and warrant further investigation.

## 1. Introduction

While commonly used as an interventional training tool for patient populations with mobility impairment [1,2,3], immersive virtual reality (VR) with head and body motion tracking also offers unique opportunities for expanding standardized assessment of physical and cognitive functioning in older adults [4,5]. Full-body tracking with accessible VR technologies are showing promise for biomechanical and clinical applications [6,7,8]. Innovative VR platforms that can conduct familiar evidence-based mobility assessments, while expanding upon the quality and quantity of information provided, can have great appeal from a clinical workflow perspective.

The ubiquitous timed up-and-go (TUG) test [9] is one such example where an already accepted and widely adopted geriatric assessment [10] has been adapted for study using immersive VR [11,12]. The TUG is a simple test that can be performed anywhere (starting from a seated position, rise and walk 3 m, turn and walk back to the chair, end in a seated position). The test observer uses a stopwatch to measure the number of seconds to complete the entire test. The clinometric properties of total TUG time are well established across multiple patient populations (see TUG entry in the Rehab Measures database https://www.sralab.org/rehabilitation-measures/timed-and-go, accessed on 10 May 2026) including dual-task versions for evaluating cognitive decline in older adults [13,14].

While useful as a global measure of mobility function, the total TUG time alone is a non-specific assessment. The TUG test is also a multi-task assessment as it includes movement sub-phases that represent different functional primitives: chair rise/sit, walking, and turning. Research into instrumented TUG (see [15] for a detailed review) has shown it is possible to partition the test into its functional primitives, inertial sensor-based motion tracking being most common [16,17,18], but, to our knowledge, no studies have examined whether VR body tracker kinematics are of sufficient quality to parameterize the TUG and its sub-phases.

As a first step to developing a comprehensive VR-based TUG test with quantitative clinical and biomechanical outcomes, the aims of our study were to answer the following questions. All other experimental conditions being equal:

1. Do participants perform the VR-based TUG test (vTUG) in the same amount of time as they perform the standard TUG (sTUG)?
2. Do participants perform the sub-tasks of the vTUG in the same amount of time as they perform the sub-tasks of the sTUG?
3. Do participants perceive the test as safe, and how do they rate the experience?

## 2. Materials and Methods

The study was reviewed and approved by the university Research Ethics Board. All participants provided informed signed consent prior to participation. The research was conducted in a university motion analysis laboratory setting between January 2021 and March 2023. Participants were recruited through local advertisement and word of mouth. Inclusion criteria were being 19 or older, willingness to try free-roaming head-mounted VR, and ability to hear and respond to instructions in English. Exclusions were any musculoskeletal or neurological conditions that affect walking, turning or getting in and out of a chair.

The vTUG tests were performed using an HTC VIVE Pro (HTC America Inc., Berkley, CA, USA) head-mounted device (HMD, 108° field of view, weight 470 g) plus seven motion trackers (HTC VIVE Trackers (2.0)) with SteamVR 1.21 (Valve Corp., Bellevue, WA, USA); five placed on the participant, one to identify the real chair location, and one to represent the 3 m turn point in VR. The vTUG condition was represented by a fully immersive virtual environment, as detailed below, which participants interacted with and provided verbal confirmation of the accuracy of the physical chair alignment with the virtual chair prior to testing.

As a preliminary study a convenience sampling approach was used. Sample size was based on an MCID (minimal clinically important difference) value for the TUG of 2 s and a Cohen effect size of 0.667, with which a sample size of 20 would be sufficient to detect at an alpha of 0.05 and a power of 0.80. Consistent with the sample method and in the spirit of caution, assuming participants would be naive to immersive VR, we first recruited and tested 10 adult participants under 65 years of age, evaluated protocol safety, and then recruited and tested 10 older adults over 65 years of age.

To guard against any possibility of injury from a fall, all participants were harnessed into an overhead robotic-controlled fall-arrest system (ZeroG™, Aretech Inc., Fairfax, VA, USA) that was set to allow for normal descent speed into a chair and minimum body weight support (5%) during ambulation. To ensure comparability, participants were harnessed for both the vTUG tests whilst wearing the VR headset and for the standard sTUG test without the VR headset. Body trackers were worn for both sTUG and vTUG tests.

### 2.1. Protocol

Participants were tested on one occasion in a university research laboratory. There were no specific instructions about clothing except that participants needed to bring indoor sneakers or walking shoes. Prior to participant arrival, the VR space was calibrated to the testing space, which included a procedure to spatially register the physical chair and the 3 m turning point on the walkway (a simple cross made with masking tape) with the virtual environment displayed to the participant. Custom software written in Unity (version 2020.3, Unity Technologies, San Francisco, CA, USA) then displayed the virtual space to the participant as a nondescript room with an armless chair and a cross on the floor 3 m from the chair.

The TUG procedure was adapted from Podsiadlo et al. [9] and Jones et al. [19] as follows. During all conditions, participants donned the ZeroG harness and five VIVE motion trackers were placed on the waist (lumbosacral region), left and right ankles (slightly superior to the lateral malleoli) and left and right wrists (slightly superior to the ulnar and radial heads). Because the HMD was wired, a research assistant was always present to assist with the cable, and participants were given an opportunity to walk through the virtual test space to verify the virtual chair and to ensure the harness and cable would not interfere in any way with task performance.

For each TUG test, participants sat comfortably on the chair, so their feet rested flat on the floor. When instructed, participants stood up from the chair and walked to the 3 m mark, turned, walked back, and turned again to sit down in the chair. Participants completed three sTUG trials and then donned the VR headset and performed three vTUG trials, with one practice test preceding each set.

After completing the TUG conditions, participants completed a post-study questionnaire (Table 1) inquiring about their experience, the quality of the VR environment, and comfortability, as well as any adverse reactions (disorientation, nausea, etc.) they may have experienced during the trials.

### 2.2. Data Analysis

Raw sensor data and the manually recorded test time during TUG trials were exported for analysis. For each body sensor, a seven-element vector was output for each time frame (100 Hz) consisting of x, y, z position and a 4-element quaternion, which were converted to Euler angles. Only three kinematic parameters from the waist sensor were required to score the total TUG and to partition it into functional sub-tasks [20]; we therefore only report these data.

**Figure 1:**
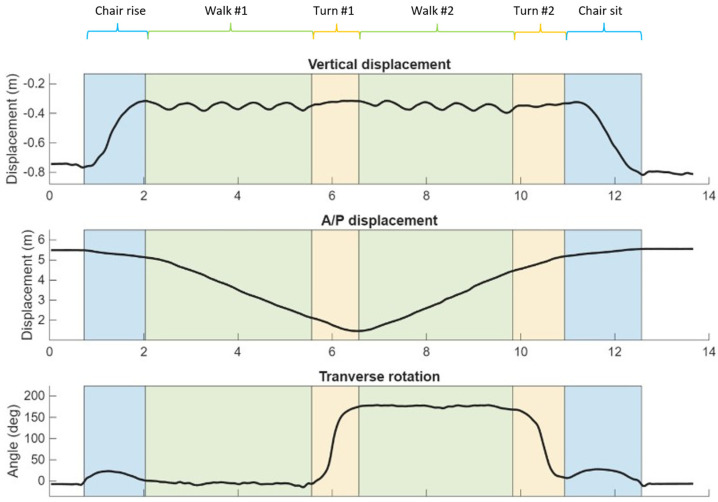
Kinematic parameters were extracted from the waist sensor of a representative subject. Vertical displacement and body rotation were used to detect the chair rise/sit and turns, respectively, which then defined the walking phases: blue = chair activity, green = walking, yellow = turning.

As shown in Figure 1, the vertical displacement (top graph) and the vertical (yaw) rotation (bottom graph) of the waist sensor were used to detect the start and end of the chair and turn portions of the test, aided by the horizontal forward displacement (middle graph) for detecting the turn point. A custom algorithm was written in Matlab (version 2023b, MathWorks, Natick, MA, USA) to auto-detect the TUG events to calculate total TUG time and the percentage of TUG time for each of the six sub-task phases: chair rise, first walk, first turn, second walk, second turn and chair sit.

To answer the first question: “Do participants perform the vTUG in the same amount of time as they perform the standard clinical TUG (sTUG)?”, we computed Intraclass Correlation Coefficients (ICCs) for absolute agreement between total TUG time scores using a two-way mixed ANOVA with averaged measures (ICC(2,k) model), for both the manual stopwatch measurement data and the automated measurement data from the computer algorithm. In addition, paired samples *t*-tests, correlation analyses and limit-of-agreement (LoA) plots were analyzed to explore measurement bias and homoscedasticity of the measures.

To answer the second question: “Do participants perform the sub-tasks of the vTUG in the same amount of time as they perform the sub-tasks of the sTUG?”, we computed ICC(2,k) coefficients to assess the agreement between the sTUG and vTUG for automated (algorithm-based) parsing of the sub-task phases. As above, *t*-tests and LoA plots were used to evaluate the quality of the sub-task interval detection. Sub-task phases were determined with a custom algorithm using the motion tracker data, allowing the trial to be partitioned into six phases: chair rise, walk #1 (to the 3 m point), turn #1 (to face the chair), walk #2 (towards the chair), turn #2 (to face away from the chair), and chair sit.

To answer the third question: “Do participants perceive the test as safe, and how do they rate the experience?”, participants completed a post-study questionnaire asking about their experience and any adverse reactions (disorientation, nausea, etc.). The survey items are summarized in Table 1. A factor analysis was conducted using a principal component analysis (PCA) with varimax rotation and Kaiser normalization to ensure (a) there was more than one source of variance in the responses, and if so (b) that independent factors will group items into interpretable domains to compare between the young adult and older adult groups. In addition, the test observer was instructed to record when any adverse events such as stumbles, loss of balance, or arrested falls occurred.

## 3. Results

Twenty healthy adults consisting of ten younger adults (five females) aged 19–36 (27.7 ± 5.7 years) and ten older adults (seven females) aged 65–71 (68.4 ± 2.5 years) participated in the study. The results from comparing the sTUG and vTUG total time scores are summarized in Table 2. Paired samples *t*-tests revealed that TUG times were approximately ½ s longer for the vTUG than for the sTUG, for both the stopwatch-measured times (*p* = 0.035) and computer-algorithm-measured times (*p* = 0.044). ICC values exceeded the threshold of acceptable agreement (>0.7) for both manual stopwatch measurement (ICC = 0.867 [95% CI: 0.636–0.949]) and computer algorithm measurement (ICC = 0.845 [95% CI: 0.592–0.940]), although it is noted that the lower bound on the 95% CI of the ICC was <0.7 for both.

**Figure 2:**
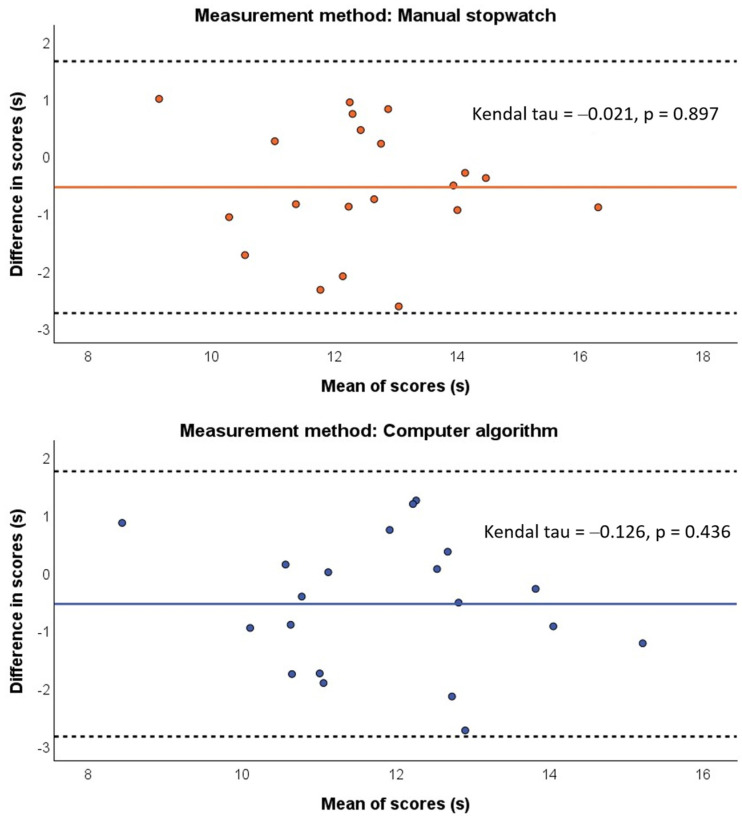
Limit-of-agreement (Bland–Altman) plots for sTUG total time compared with vTUG total time. The horizontal axis is the mean of the two measures ([sTUG time + vTUG time]/2), and the vertical axis is the difference between the two measures (sTUG time − vTUG time). The horizontal dashed lines represent +/− 2*SD from the mean. Kendall’s tau correlations indicate the threat of heteroscedasticity is very low.

Comparison of manual stopwatch time and test times determined by the computer algorithm operating on motion tracker sensor data are also shown in Table 2. Stopwatch times were on average 0.6 s longer than test times from the computer algorithm (*p* < 0.001). The ICC values for absolute agreement were high for both sTUG (ICC = 0.927 [95% CI: 0.545–0.979]) and vTUG (ICC = 0.953 [95% CI: 0.391–0.988]), we again note the lower-bound on the ICC was <0.7. As shown in Figure 2, the LoA plots indicate the measures are homoscedastic with non-significant Kendall’s tau correlations.

The results of the sub-task phase (% of total TUG time) scores are summarized in Table 3. Chair rise duration, first turn, and second walk had ICC values > 0.7, while first walk, second turn and chair sit had ICC values < 0.7, with the last turn and sit each having the lowest ICCs with 95% CI enclosing zero. Combining the final turn and sit phases improved the ICC value for this sub-task, as shown in the bottom row of Table 3.

There were zero adverse events reported in the form of stumbles, loss of balance or arrested falls (from the overhead ZeroG system). Overall, the majority (>50%) of participants tended toward the highest positive response for all questions. To further explore responses, PCA identified three distinct domains: experience in VR environment, quality of VR environment, and comfort of testing apparatus. All satisfaction scores were above 80% for both young and older adults, except for comfort; there was a statistically significant difference in comfort by age, where the young adult group rated comfort lower than older adults. Results of the participant experience survey are shown in Table 4.

## 4. Discussion

Immersive head-mounted VR offers unique opportunities for extracting considerable value from common tests of physical functioning, such as the ubiquitous TUG test, without requiring a motion analysis laboratory. The VR environment allows for standardization of test scenarios and the introduction of novel distractor elements such as mental challenges, virtual obstacles, and other forms of dual-task interference testing for assessing cognitive decline in older adults and patient populations [11,13,16,21].

A motivation for studying the TUG is that performing the task requires executing key functional movements—rising from and sitting on a chair, walking, and turning. The typical clinical TUG, however, is only scored based on total time and typically with a manual timing device (stopwatch, smartphone, etc.). Regardless of the TUG testing paradigm, the ability to automate total and sub-task (chair, walking, and turning) performance during the TUG test, whether conducted with head-mounted device (vTUG) or without (sTUG), is an inexpensive value-added feature of VR body tracking systems.

The rapid adoption and affordability of head-mounted VR and synchronized body motion tracking enable sensor-based acquisition of movement data during the examination, allowing for the simple TUG test outcomes to be expanded to performance indicators of these key functional movements. Previous studies employing inertial measurement units (accelerometers, gyroscopes, etc.) during the TUG [16,22] have already demonstrated the added value of including sensors as part of the protocol, but, to date, widespread clinical adoption of inertial-measurement-unit (IMU) technology has not happened. The motion trackers available with VR systems are tracked as rigid bodies in a calibrated space and therefore do not suffer the complexities of integrating multi-DOF IMU signals.

Our study demonstrated that an off-the-self VR system with a custom designed interface built in Unity could be used to conduct a virtual TUG test that faithfully assesses the same underlying functional substrates as the standard clinical TUG, with the added value of enabling automatic quantification of test time and movement sub-task time (e.g., in % of total TUG time) and ability to standardize the testing environment regardless of where the test is done. While not analyzed in this paper, the extremity motion trackers also add value by potentially enabling spatiotemporal gait parameters such as stride length and width, stride time, speed and cadence to be acquired in any environment without the need for cameras or IMUs.

Although the test results for the sTUG and vTUG were highly congruent, there were some differences observed that required comment. First, there appeared to be a relatively consistent offset between the total TUG time for the two tests, with the vTUG taking approximately ½ second longer than the sTUG. This agrees with other studies that have suggested a possible VR effect to explain decrements in performance in vTUG compared with standard testing [12,23,24]. It is worth noting that a difference of ½ sec is considerably smaller than the minimal detectable change (over 4 s for Alzheimer’s disease and related dementia) reported for the TUG [21]. In other words, although the scoring offset was statistically significant, the difference may not actually be important, which probably explains why the ICC result for absolute agreement was very high (>0.85), but, as noted, the 95% CI on the ICC had a lower bound that fell below 0.7.

Examination of the sub-task scores did little to explain the observed difference in total time. In fact, there was no one sub-task where the vTUG took significantly longer (had a higher % of total TUG time) than the sTUG; rather, there seemed to be small decrements in gait and final turn and sit time, but curiously, the first turn relative time (% of total TUG time) was significantly lower (meaning faster relative to total time) for the vTUG compared with the sTUG (*p* = 0.038). A possible explanation is the lack of randomized order of vTUG and sTUG, where the participant always completed sTUG prior to vTUG.

Comparison of the sub-task time proportions to a gold standard was not possible in the current study; however, our results, including the combination turn and sit times agree very well with several published studies that have used a variety of motion capture methods, from marker-based optical tracking to inertial sensors, to partition the TUG in young and older participants [20,22,25]. For example, even though turn and sit time had lower ICC values, the magnitude (23.9% of the TUG) was similar to data for healthy participants using inertial sensors (24.8% of the TUG in [20]). It is likely the low ICCs for second turn and stand-to-sit are a consequence of difficulty demarking a consistent transition point; i.e., healthy individuals may start to descend into the seat while turning. As such, combining the last two phases into a single “turn-and-sit” phase is probably better and more functionally relevant.

PCA was used to determine whether there was an underlying structure to participants’ responses on the VR satisfaction survey. Although the sample was small, three clearly separate domains emerged: experience in VR, quality of the VR, and comfort of the VR equipment. Satisfaction scores were above 80%, except for comfort which was significantly lower for younger adults than for older adults (*p*-value = 0.025). Younger participants who volunteered early in the study likely experienced more issues with the HMD cable during turns (i.e., being cognizant of which way to turn), which was addressed well before testing older adults. The PCA analysis also suggests that the quality of the experience was predicated by the strength of their trust that an actual chair existed while in the VR condition.

The study had limitations which need to be considered when interpreting the findings. The ZeroG system was implemented to eliminate any possibility of a fall related injury, particularly in the older adult cohort who would be naive to head-mounted overground VR. Because the study proved to be safe and no adverse events occurred, these precautions will not be required for future study of the vTUG. Other physical restrictions include the wired HMD which required practice on behalf of the participant and diligence on behalf of the tester. Replicating this study with a wireless HMD and without a fall arrest harness is plausible and recommended to increase comfort, operability and ecological relevance.

As another measure of safety, participants were not assumed to have any prior knowledge or experience having a TUG assessment, and therefore the decision was made to have all participants complete the sTUG prior to the vTUG. This may have resulted in practice effects that could have influenced the first turn, for example. However, as noted in the Results and Discussion these differences were very small and not likely important.

Another possible limitation was the lack of a virtual avatar to reflect the participants’ movements in VR. Avatar representation of the extremities is important for tasks involving biofeedback such as upper limb prosthetic training [26] or post-stroke rehabilitation [27], for example. Although visual feedback was not deemed necessary for the purpose of our comparative study in healthy participants, for future research with more-complex mobility and spatial awareness tasks, implementing an avatar may be necessary.

Finally, the inclusion of a broad age range was in part a product of including the younger cohort as a measured approach to safe protocol design for older adults. While less important clinically to include younger adults, the pooled data for ICC analysis resulted in a wider spread of TUG scores, which likely explained in part the high ICCs and narrow confidence intervals. While power may have been sufficient for testing the primary outcome, total TUG time, the lower ICCs on some of the sub-phase intervals may require more-robust a priori powering [28] of other methods for assessing concordance such as the Passing–Bablok regression [29].

## 5. Conclusions

This study’s results support that the vTUG and sTUG tests reflect functional similarity, evident through the similarities in time to complete the segmented portions of the TUG tests. This segmentation is possible through motion tracker data provided by the HMD and trackers. Larger samples might yield significant differences between standard and VR testing results, as the difference scores were biased toward longer VR TUG times. Because this effect was seen in both the manual and automated computer measurements, the bias was not likely due to operator error (such as needing to pay attention to more things when the participant was wearing the VR headset) and therefore could indicate that participants who are naïve to the VR testing may move slightly slower than when performing the test without the VR headset.

Lack of adverse events during data collection supports future testing without the safety assurance provided by the BWS system.

## Figures and Tables

**Table 1:** Questions on the VR satisfaction survey.

Q1: When wearing the VR-HMD, how well did the virtual chair correspond to the actual chair?	Not very well
	Well enough
	Very well
Q2: Did you experience any disorientation when wearing the HMD?	A lot
	A little
	None at all
Q3: Did you experience any nausea when wearing the HMD?	A lot
	A little
	None at all
Q4: Was the HMD comfortable to wear during the experiment?	Some of the time
	Most of the time
	All of the time
Q5: Were the trackers comfortable to wear during the experiment?	Some of the time
	Most of the time
	All of the time
Q6: Was this experience enjoyable?	No
	Neutral
	Yes

**Table 2:** vTUG compared with sTUG for both measurement methods by row, and comparison between measurement methods by column.

	TUG Test ^†^				Paired Comparisons			
Meas. Method	sTUG		vTUG		Diff.	SD	*t*-Test *, *p*	ICC ^‡^ (95% CI)
	Mean	SD	Mean	SD				
**Stopwatch**	12.21	1.67	12.76	1.76	−0.55	1.09	0.035	0.867 (0.636−0.949)
**Algorithm**	11.60	1.58	12.15	1.75	−0.55	1.15	0.044	0.845 (0.592−0.940)
**Diff.**	0.61	0.64	0.61	0.48				
***t*** -test *, *p*	<0.001		<0.001					
**ICC ^‡^** **(95% CI)**	0.927 (0.545–0.979)		0.953 (0.391–0.988)					

^†^ sTUG = standard TUG; vTUG = virtual TUG. * Significance of paired samples *t*-test. ^‡^ Intraclass Correlation Coefficient (ICC) model (2,k) for absolute agreement.

**Table 3:** Analysis results of TUG sub-tasks phases (% of total TUG time) for the virtual TUG (vTUG) compared with standard TUG (sTUG). Note, the final row “Turn and sit” is “Turn #2” and “Chair sit” as a singular task.

	TUG Test ^†^				Paired Comparisons			
TUG Phase (% Total TUG Time)	sTUG		vTUG		Diff.	SD	*t*-Test *, *p*	ICC ^‡^ (95% CI)
	Mean	SD	Mean	SD				
Chair rise	9.06	3.22	8.79	2.85	0.27	2.27	0.595	0.842 (0.601−0.938)
Walk #1	28.98	3.92	29.74	2.82	−0.76	3.62	0.362	0.612 (0.029−0.846)
Turn #1	8.42	2.84	7.31	2.11	1.10	2.21	0.038	0.723 (0.307−0.890)
Walk #2	29.61	3.79	30.23	3.61	−0.62	3.42	0.430	0.733 (0.328−0.894)
Turn #2	7.83	1.83	7.69	2.08	0.14	2.20	0.781	0.551 (−0.170−0.825)
Chair sit	16.10	3.37	16.25	2.90	−0.15	3.87	0.868	0.406 (−0.573−0.769)
Turn & sit	23.93	3.92	23.93	3.39	−0.01	3.66	0.993	0.678 (0.165−0.874)

^†^ sTUG = standard TUG; vTUG = virtual TUG. * Two-tailed paired samples *t*-test. ^‡^ Intraclass Correlation Coefficient for absolute agreement between measures.

**Table 4:** Satisfaction scores (0–100): experience, quality, and comfort were compared between the young and older adults with a 2-tailed independent samples *t*-test.

	Age Group	N	Mean	SD	t (df)	*p*-Value
Experience	Young adult	10	90.0	12.9	−0.949 (18)	0.355
	Old adult	10	95.0	10.5		
Quality	Young adult	10	92.5	12.1	0.739 (18)	0.470
	Old adult	10	87.5	17.7		
Comfort	Young adult	10	75.0	23.6	−2.449 (18)	0.025
	Old adult	10	95.0	10.5		

## Data Availability

The raw data supporting the conclusions of this article will be made available by the authors on request.

## Abbreviations

The following abbreviations are used in this manuscript:

vTUG	Virtual timed up-and-go (TUG) test
sTUG	Standard TUG test
ICC	Intraclass correlation coefficient
LOAs	Limits of agreement
